# Triosephosphate isomerase from *Fasciola hepatica*: high-resolution crystal structure as a drug target

**DOI:** 10.1107/S2053230X25006454

**Published:** 2025-08-20

**Authors:** Georgios Kontellas, David J. Studholme, Mark van der Giezen, David J. Timson, Jennifer A. Littlechild, Michail N. Isupov

**Affiliations:** a Henry Wellcome Building for Biocatalysis, Biosciences, Faculty of Health and Life Sciences University of Exeter ExeterEX4 4QD United Kingdom; b Biosciences, Faculty of Health and Life Sciences University of Exeter ExeterEX4 4QD United Kingdom; c Centre for Organelle Research, Department of Chemistry, Bioscience and Environmental Engineering University of Stavanger 4021Stavanger Norway; d School of Pharmacy and Biomolecular Sciences University of Brighton BrightonBN2 4GJ United Kingdom; University of York, United Kingdom

**Keywords:** *Fasciola hepatica*, triosephosphate isomerase, TPI, TIM, triclabendazole, crystal structure

## Abstract

The trematode liver fluke *Fasciola hepatica* causes the neglected tropical disease fascioliasis in humans and is associated with significant losses in agricultural industry due to reduced animal productivity. Triosephosphate isomerase (TPI) is a glycolytic enzyme that has been researched as a drug target for various parasites, including *F. hepatica*. The high-resolution crystal structure of *F. hepatica* TPI (*Fh*TPI) has been solved at 1.51 Å resolution in its monoclinic form. The structure has been used to perform molecular-docking studies with the most successful fasciolocide triclabendazole (TCBZ), which has recently been suggested to target *Fh*TPI. Two *Fh*TPI residues, Lys50 and Asp51, are located at the dimer interface and are found in close proximity to the docked TCBZ. These residues are not conserved in mammalian hosts.

## Abstract 2

The high-resolution crystal structure of triosephosphate isomerase from *F. hepatica* was solved at 1.51 Å resolution in its monoclinic form, revealing details of the dimer interface critical for enzyme function. Molecular docking with the fasciolocide triclabendazole suggests selective binding near nonconserved residues, highlighting this enzyme as a promising parasite-specific drug target.

## 1. Introduction

*Fasciola hepatica*, the common liver fluke, is a digenean trematode belonging to the phylum Platyhelminthes. It causes a zoonotic disease described as fascioliasis in humans and as fasciolosis in herbivores by infecting the liver (Seah, 1978 ▸). It has been included by the World Health Organization (WHO) in its catalogue of neglected tropical diseases under the subset of foodborne tremadodiases (World Health Organization, 2013 ▸). More than 2.4 million people in at least 75 countries and across five continents have been estimated to be infected worldwide (World Health Organization, 2020 ▸). *F. hepatica* disease shows the widest global distribution among the foodborne diseases (Mas-Coma *et al.*, 2019 ▸) and climate change is contributing to its further spread (Fox *et al.*, 2011 ▸; Booth, 2018 ▸).

While the juvenile flukes mature inside their mammalian host, they progressively depend on anaerobic metabolism due to the very low availability of oxygen (Tielens, 1994 ▸). As a result, the adult flukes rely almost entirely on glycolysis and an anaerobically functioning electron-transport chain (Tielens *et al.*, 1984 ▸; Lloyd, 1986 ▸; Tielens & Van Hellemond, 1998 ▸).

Triosephosphate isomerase (TPI; EC 5.3.1.1) is a glycolytic enzyme which catalyses a critical step in glycolysis in which a toxic triose dihydroxyacetone phosphate (DHAP; produced by aldolase in the previous step) is reversibly converted to d-glyceraldehyde-3-phosphate (GAP; Rieder & Rose, 1959 ▸). GAP is further processed in glycolysis, highlighting its importance in central energy metabolism. Thus, whenever there is influx of DHAP from the previous steps of glycolysis, it can be isomerized to GAP and further processed to pyruvate (Boiteux & Hess, 1981 ▸).

The first TPI structure was determined from chicken muscle (Banner *et al.*, 1975 ▸). The TPI structure is a typical example of an α/β barrel, formed of α-helices and β-sheets, which is one of the most common motifs found in proteins (Brändén, 1991 ▸; Hegyi & Gerstein, 1999 ▸). The TPI, in most species, is a homodimer with the substrate-binding site located close to the dimer interface (Jogl *et al.*, 2003 ▸; Velanker *et al.*, 1997 ▸; Mukherjee *et al.*, 2012 ▸; Delboni *et al.*, 1995 ▸; Wierenga *et al.*, 1987 ▸; Parthasarathy *et al.*, 2003 ▸; Alber *et al.*, 1981 ▸). Glycolytic enzymes have always been considered to be attractive drug targets in helminths (Timson, 2016 ▸). There are several examples of compounds that selectively inhibit TPI in unicellular parasitic eukaryotes (Enriquez-Flores *et al.*, 2008 ▸, 2011 ▸; Gayosso-De-Lucio *et al.*, 2009 ▸; Olivares-Illana *et al.*, 2006 ▸, 2007 ▸; Maithal *et al.*, 2002 ▸; Rodríguez-Romero *et al.*, 2002 ▸) that could be significant for the development of successful chemotherapies.

The TPI from *F. hepatica* (*Fh*TPI) has been biochemically characterized (Zinsser *et al.*, 2013 ▸) and its structure has been reported at a resolution of 1.9 Å in a trigonal space group (Ferraro *et al.*, 2020 ▸). The latter study also proposed that *Fh*TPI is inhibited by the most widely used fasciolocide, triclabendazole (TCBZ), with an IC_50_ of 7 µ*M*.

Of special interest is the dimer interface of the enzyme. Only the dimeric form of TPI is active, and disruption of the interactions forming the dimer can result in inhibition and inactivation of the enzyme (Téllez-Valencia *et al.*, 2002 ▸). TPI dimer-interface inactivators have been investigated in the past with promising results. Three compounds demonstrated selective inhibitory effects against TPI from *Trypanosoma cruzi* both *in vitro* and *in vivo* (Aguilera *et al.*, 2016 ▸). The TPI from the cattle tick *Rhipicephalus microplus* was shown to be inhibited with IC_50_ values of between 25 and 50 µ*Min vivo* (Saramago *et al.*, 2018 ▸). Both studies strengthen our hypothesis that *Fh*TPI could be exploited as a molecular drug target.

In this study, we report the expression, purification and crystallization of *Fh*TPI and its monoclinic structure at 1.51 Å resolution. As attempts to crystallize *Fh*TPI in complex with the potential inhibitor TCBZ did not yield any diffraction-quality crystals, the structure was used in molecular-docking experiments to locate structural differences between parasitic and mammalian host enzymes, with the aim of developing new parasite-specific inhibitors of glycolysis. The docking results suggest that TCBZ binds close to the dimer interface and proximate to two residues that form hydrogen bonds and salt bridges that stabilize it. Disturbance of these interactions could increase the understanding of the specific inhibitory effect of TCBZ.

## 2. Materials and methods

### 2.1. Protein overexpression and purification

Plasmid pET-46 Ek/LIC, derived from pRT-46 (Merck, Darmstadt, Germany) and containing the gene for *Fh*TPI, was kindly provided by Professor David Timson (University of Brighton, UK). This expression vector adds an N-terminal histidine tag (MAHHHHHHDDDDK) onto the protein for ease of purification. This construct was transformed into *Escherichia coli* Rosetta (DE3) competent cells (Merck, Darmstadt, Germany). Single colonies were grown on Luria–Bertani medium at 310 K to an optical density of 0.6 at 600 nm. The cultures were then induced with 1 m*M* isopropyl β-d-1-thiogalactopyranoside for 3 h. The cells were harvested by centrifugation at 4700*g* for 30 min at 277 K. The harvested cells were resuspended in buffer *A* (50 m*M* Tris–HCl, 300 m*M* NaCl, 10 m*M* imidazole pH 7.0) and lysed by sonication after ten cycles of 30 s (with 30 s rest on ice between cycles).

The soluble fraction was collected after centrifugation at 24 000*g* at 277 K for 30 min and loaded onto a 1 ml HisTrap FF Crude immobilized metal-affinity chromatography (IMAC) column (GE Healthcare, Cincinnati, USA) pre-equilibrated with buffer *A*. Unbound protein was washed from the column with six column volumes of buffer *A*. Bound protein was eluted with a 20-column-volume gradient to 100% buffer *B* (50 m*M* Tris, 300 m*M* NaCl, 250 m*M* imidazole pH 7.0) at a 1 ml min^−1^ flow rate. Fractions corresponding to recombinant *Fh*TPI were pooled and loaded onto a 120 ml HiLoad 16/600 Superdex 200 pg size-exclusion chromatography (SEC) column (GE Healthcare, Cincinnati, USA) and eluted under isocratic conditions with two column volumes of SEC buffer (25 m*M* Tris, 300 m*M* NaCl pH 7.0) at a flow rate of 0.8 ml min^−1^. The purification stages were performed at 277 K and the protein fractions were analysed by SDS–PAGE. Protein concentration was calculated by measuring the absorbance at 280 nm using a NanoDrop 2000c (Thermo Fisher Scientific, Massachusetts, USA). All purification steps were performed with an ÄKTApurifier system (GE Healthcare, Cincinnati, USA).

### 2.2. Crystallization

Microbatch 96-well crystallization plates (Douglas Instruments, Berkshire, UK) were used for crystal screening with an Oryx8 crystallization robot (Douglas Instruments, Berkshire, UK). The protein was concentrated to a concentration of 15 mg ml^−1^ using an Amicon Ultra-15 Centrifugal Filter Unit with a 10 kDa molecular-weight cutoff (Merck, Darmstadt, Germany). The protein was screened with the following commercial crystallization screening kits (Molecular Dimensions, Newmarket, UK): JCSG-plus, The Stura Footprint Combination, Morpheus and MultiXtal. The final droplet consisted of 0.5 µl protein solution and 0.5 µl crystallization screen. Droplets were covered with Al’s oil (a 1:1 ratio of silicone and paraffin oil) and incubated at 291 K. During the incubation period, droplets were frequently checked under a light microscope.

The set of conditions that produced the crystal that diffracted to the highest resolution were 0.2 *M* Li_2_SO_4_, 0.1 *M* ADA {2,2′-[(2-amino-2-oxoethyl)imino]diacetic acid} pH 6.5, 30%(*v*/*v*) PEG 400.

### 2.3. X-ray data collection

Crystallographic data were collected on the I02 beamline at the Diamond Light Source (Didcot, UK) at 100 K in a stream of gaseous nitrogen using a PILATUS 6M-F X-ray detector (Dectris, Baden-Daettwil, Switzerland). Data were processed and scaled using *XDS* (Kabsch, 2010 ▸) and *AIMLESS* (Evans & Murshudov, 2013 ▸) in the *xia*2 pipeline (Winter *et al.*, 2013 ▸). Further data and model manipulations were performed using the *CCP*4 software suite (Agirre *et al.*, 2023 ▸).

### 2.4. Structure solution and refinement

Utilizing the *MoRDa* automatic molecular-replacement pipeline (Vagin & Lebedev, 2015 ▸), the structure of *Fh*TPI was successfully solved. The model template chosen by *MoRDa* was the dimeric triosephosphate isomerase model from *Homo sapiens* (PDB entry 2jk2; Rodríguez-Almazán *et al.*, 2008 ▸). The *Coot* software (Emsley *et al.*, 2010 ▸) was used for manual rebuilding of the model. Refinement of the model was performed with *REFMAC* version 5.8.0135 (Murshudov *et al.*, 2011 ▸) with input of external phases (Pannu *et al.*, 1998 ▸) from density modification including fourfold averaging in *Parrot* (Cowtan, 2010 ▸). After refinement, the quality of the structure was analysed using *MolProbity* (Williams *et al.*, 2018 ▸). The atomic coordinates and structure factors have been deposited in the Protein Data Bank with accession code 7qon.

### 2.5. Molecular docking

Docking simulations of ligands were performed using *AutoDock* 4.2 and its accompanying interface *AutoDockTools* version 1.5.6 (Morris *et al.*, 2009 ▸). The solved high-resolution crystal structure of *Fh*TPI was used as the receptor and TCBZ as the ligand in these molecular-docking studies. Both molecules were prepared with *AutoDockTools* by adding polar hydrogens, merging nonpolar hydrogens, computing the Gasteiger atomic charges and identifying and assigning the possible rotatable bonds of each ligand. Within the parameters of the Lamarckian genetic algorithm, the maximum number of energy evaluations (25 000 000, long option) was chosen to perform the conformational searching at the highest possible accuracy. Two experiments were performed, the first searching the entire surface of the dimer and the second searching the dimer-interface area. The search grid of *AutoDock* was configured accordingly to include the areas of interest. All resulting poses were examined visually with *PyMOL* (Schrödinger).

## 3. Results and discussion

### 3.1. Overexpression, purification and crystallization

**Figure 1:**
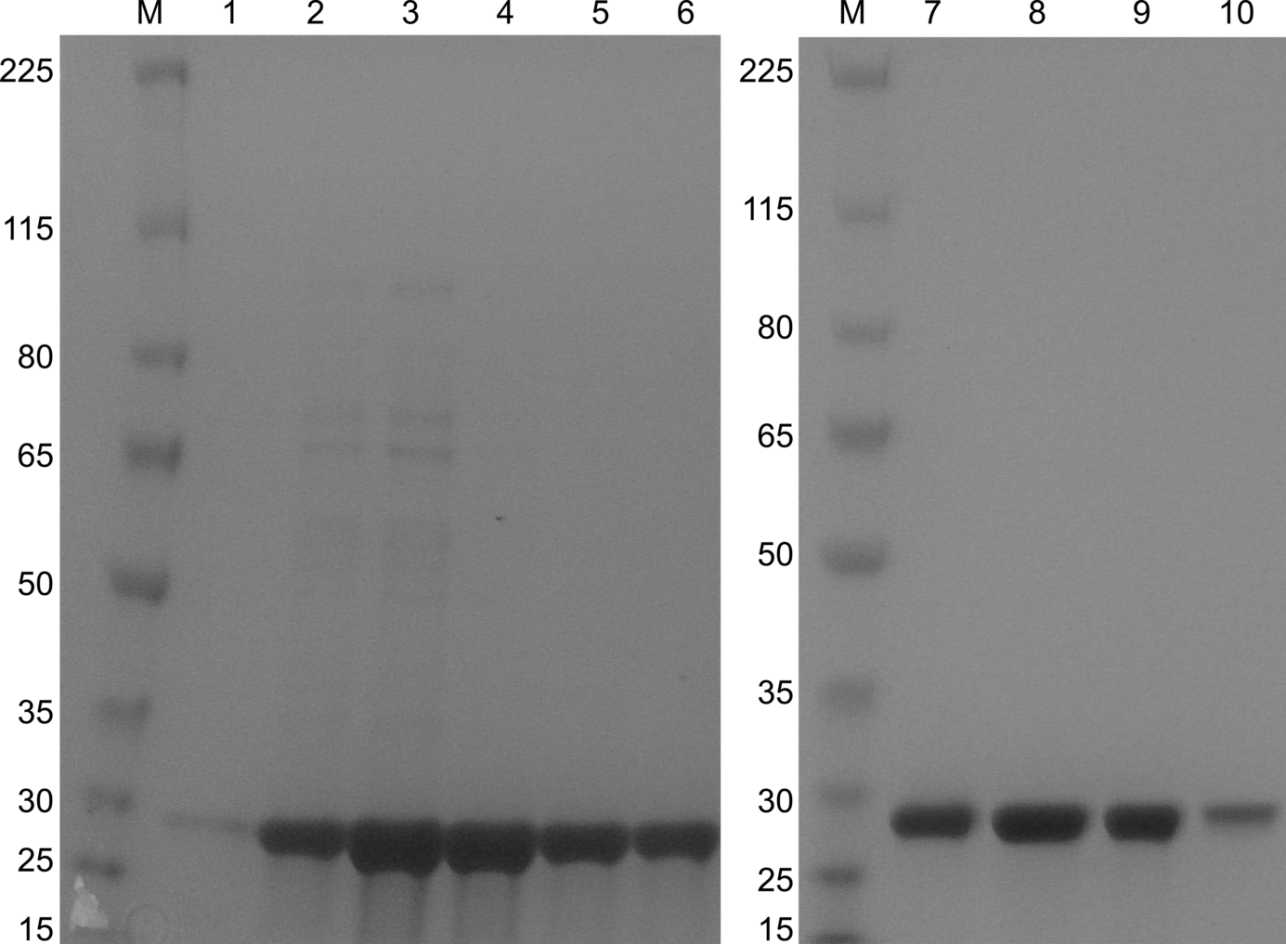
SDS–PAGE gels of the eluted fractions from the IMAC column and the HiLoad 16/600 Superdex 200 pg SEC column. Lane *M*, molecular-weight marker (kDa). Lanes 1, 2, 3, 4, 5 and 6, *Fh*TPI IMAC fractions along the elution peak. Lanes 7, 8, 9 and 10, *Fh*TPI SEC fractions along the elution peak.

The *Fh*TPI construct was successfully overexpressed in *E. coli* strain Rosetta (DE3). Subsequently, the soluble fraction of the protein was purified by IMAC. The *Fh*TPI migrated on SDS–PAGE as a single band of approximately 30 kDa (Fig. 1 ▸). The IMAC-purified fractions were collected and were further purified by SEC, where the protein eluted at a volume corresponding to a molecular weight of 60 kDa, suggesting that *Fh*TPI is a homodimer in solution.

### 3.2. Model quality

**Figure 2:**
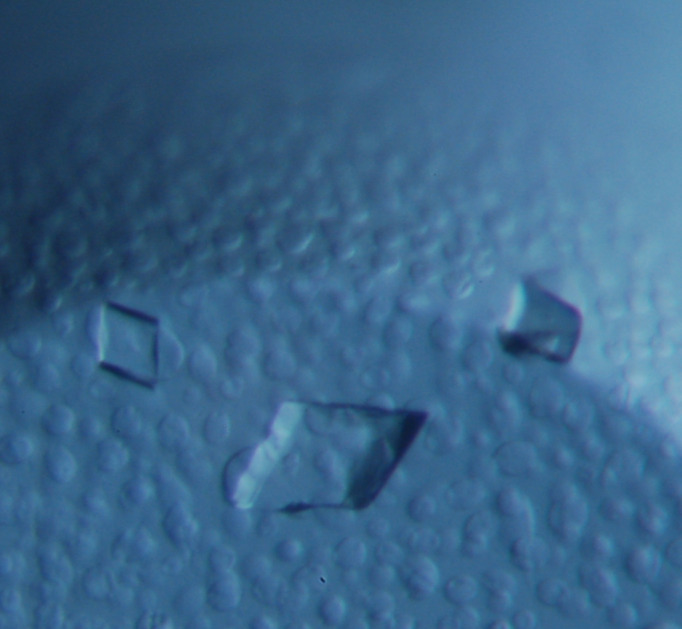
The monoclinic crystal of *F. hepatica* triosephosphate isomerase.

The best-diffracting crystal was grown in 0.2 *M* Li_2_SO_4_, 0.1 *M* ADA pH 6.5, 30%(*v*/*v*) PEG 400, with dimensions of 0.3 × 0.2 mm (Fig. 2 ▸). This crystal diffracted to 1.51 Å resolution and belonged to the monoclinic space group *P*12_1_1 with unit-cell parameters *a* = 42.40, *b* = 110.47, *c* = 118.13 Å, α = 90, β = 97.38, γ = 90°. The asymmetric unit contains two *Fh*TPI dimers. The MR solution was rebuilt and the resulting model was refined to acceptable *R* factors and stereochemical parameters (Table 1 ▸). Asp35 is a Ramachandran outlier in each of the subunits. In subunit *C*, several loops were built with an alternative conformation of the main chain.

### 3.3. *Fh*TPI structure

**Figure 3:**
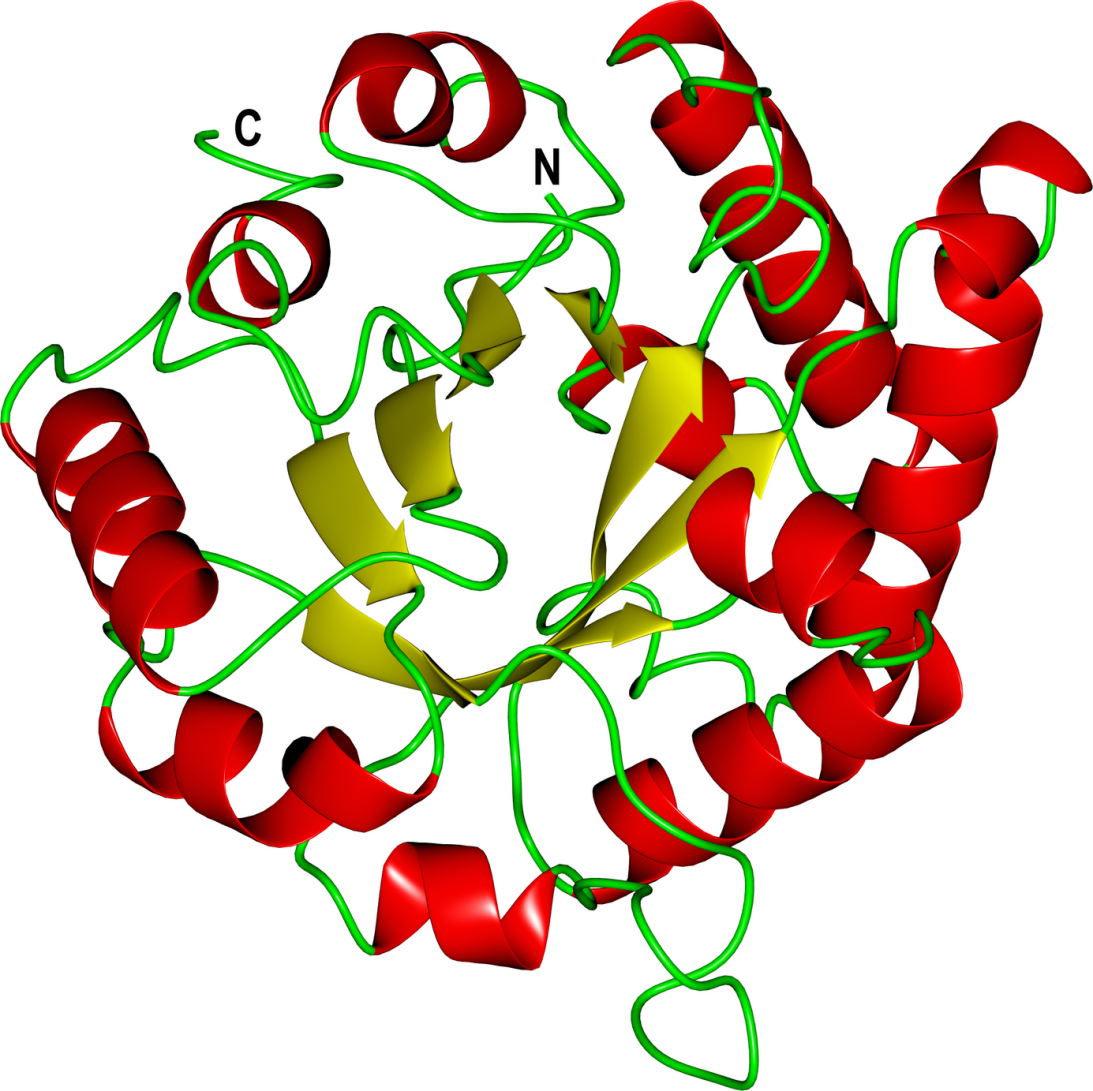
Tertiary structure of the *Fh*TPI monomer in a cartoon representation. The α-helices are coloured red, β-strands yellow and loop regions green. This figure was produced with *CCP*4*mg* (McNicholas *et al.*, 2011 ▸).

**Figure 4:**
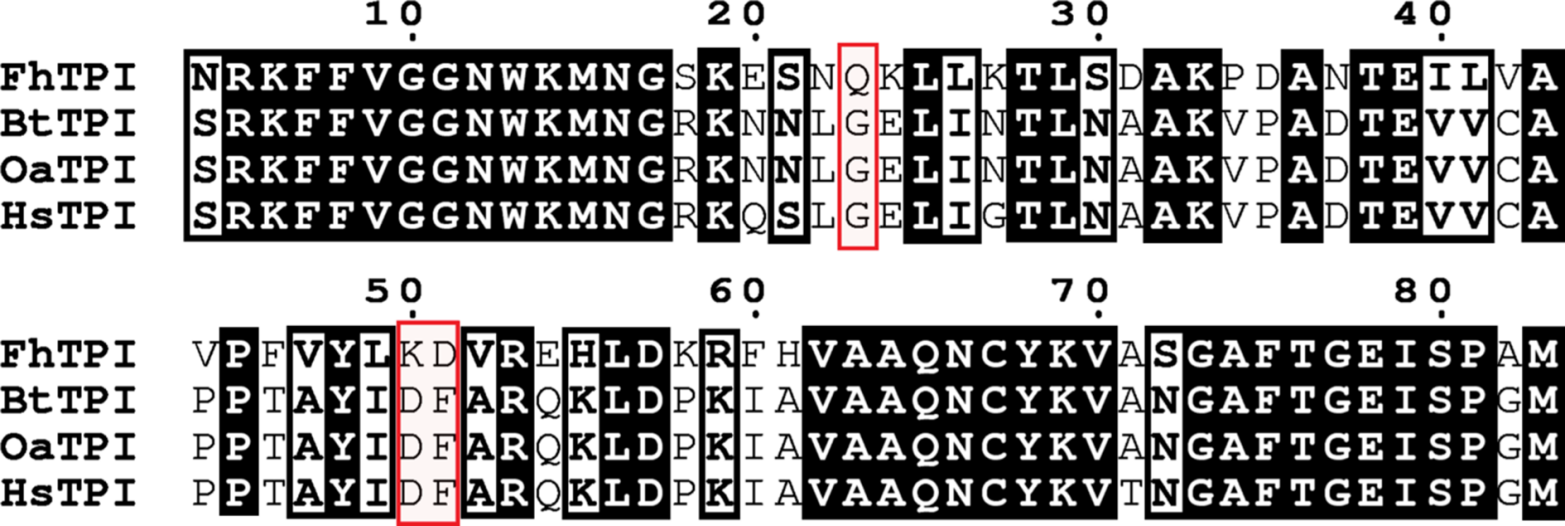
Fragment of the multiple sequence alignment of TPI amino-acid sequences from *F. hepatica* (Swiss-Prot entry S4UI50), *B. taurus* (Swiss-Prot entry Q5E956), *O. aries* (UniProt entry A0A6M6R7Y5) and *H. sapiens* (Swiss-Prot entry P60174). Residues of interest are framed in red boxes. Shaded residues represent identical residues. Residues with similar physicochemical properties are framed in black boxes. Alignment was performed with *Clustal Omega* (Sievers *et al.*, 2011 ▸). The figure was produced using *ESPript*3 (Robert & Gouet, 2014 ▸).

Analysis of the secondary structure by *PROMOTIF* (Hutchinson & Thornton, 1996 ▸) using the EBI *PDBsum* server (Laskowski *et al.*, 2018 ▸) reveals that the monomer structure consists of 14% β-strands and 37.2% α-helices. The main secondary structures of the *Fh*TPI monomer are a single β-sheet (an eight-stranded barrel) and 15 α-helices, which are arranged in a α/β barrel motif. This is one of the most common and widespread folds (observed in many enzyme families) found in proteins (Hegyi & Gerstein, 1999 ▸). Fig. 3 ▸ illustrates, with a cartoon, the secondary structure of the *Fh*TPI monomer. Analysing the crystal structure of *Fh*TPI further using the *PDBePISA* (*Protein Interfaces, Surfaces and Assemblies*) service at the European Bioinformatics Institute (https://www.ebi.ac.uk/msd-srv/prot_int/pistart.html; Krissinel & Henrick, 2007 ▸) reveals that 39 residues of each monomer participate in formation of the dimer interface. There are 27 hydrogen bonds and seven salt bridges at the dimer interface that stabilize it. Out of these interactions, of especial interest is the interaction of Lys50 and Asp51 that participate in hydrogen-bond and salt-bridge formation at the dimer interface. These two residues do not exist in the mammalian hosts of *F. hepatica*, where they are substituted by Asp50 and Phe51 as shown by the aligned TPI sequences (Fig. 4 ▸). Analysis of the mammalian host TPI structures with *PDBePISA* shows that Phe51 does not participate in dimer-forming bonds. The *Plasmodium falciparum* TPI (*Pf*TPI) crystal structure has the ligand 3-phosphoglycerate (3-PG) bound to the dimer interface as well as the active site (Gayathri *et al.*, 2009 ▸). 3-PG, a substrate analogue of TPI, exhibited weak inhibition of *Pf*TPI with a *K*_i_ of 2 m*M*. This finding is in agreement with our docking results, suggesting that the dimer interface can be a site where small molecules can bind and produce allosteric effects on TPI.

In this study, the *Fh*TPI structure has also been used for molecular-docking studies. We chose to perform these simulations as crystallization attempts to produce TPI structures complexed with inhibitors and substrates were unsuccessful. The ligand we chose to use is the flukicide TCBZ, which has been proposed to inhibit *Fh*TPI (Ferraro *et al.*, 2020 ▸). We performed two docking experiments with different approaches, where in the first the whole dimer was used as the docking area (also called ‘blind’ docking) and in the second the ligand was docked on the dimer-interface area (‘targeted’ docking). Ferraro *et al.* (2020 ▸) conducted both ‘blind’ docking across the entire surface of the *Fh*TPI monomer and dimer and ‘targeted’ docking focused on the active site, but did not specifically explore the dimer interface as in our study. In contrast, our docking simulations specifically targeted the dimer-interface region, whereas Ferraro *et al.* (2020 ▸), to our understanding, conducted their ‘targeted’ docking on the monomeric form. This methodological difference may account for the distinct binding location of TCBZ observed in our experiments. In our studies, both the ‘targeted’ and ‘blind’ docking approaches identified TCBZ binding poses in a region within the dimer interface, distant from the catalytic residues. Furthermore, our analysis revealed that Lys50 and Asp51, which are involved in dimer-interface formation, are not conserved in the mammalian hosts of *F. hepatica*, with potential selectivity implications.

**Figure 5:**
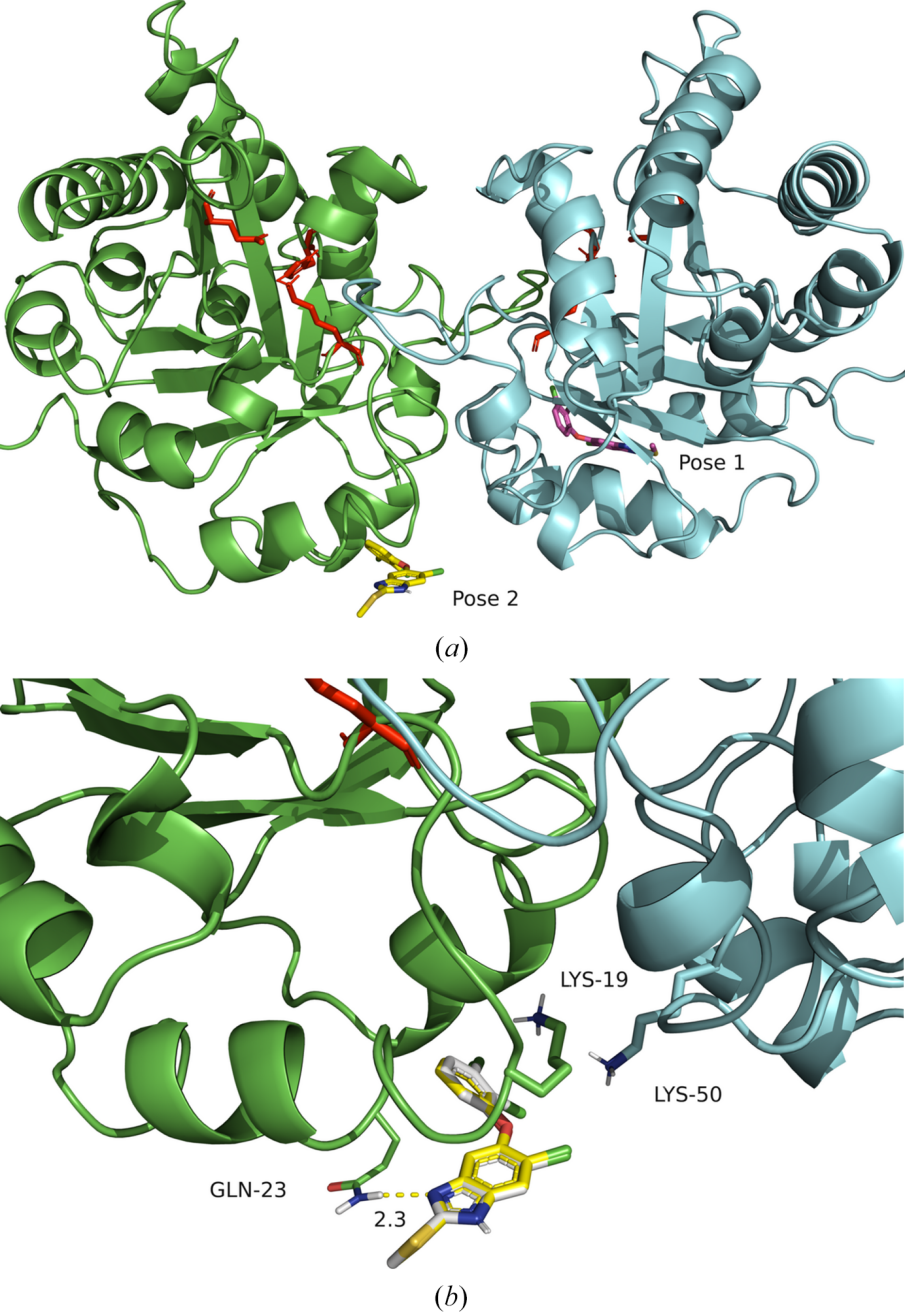
(*a*) Front view of the *Fh*TPI dimer with the best and second-best docking poses of TCBZ displayed (magenta and yellow, respectively). The whole dimer was used as the docking area. Active-site residues for each monomer are shown as red sticks. (*b*) Close-up view of the second- and third-best docking poses (yellow and grey, respectively) showing TCBZ docked close to the *Fh*TPI dimer interface. Residues (participating in the dimer interface) of both monomers within a radius of 4 Å from TCBZ are displayed. The yellow dashed line indicates (in Å) the predicted hydrogen bond between Gln23 and TCBZ. This image was produced with *PyMOL* (Schrödinger).

**Figure 6:**
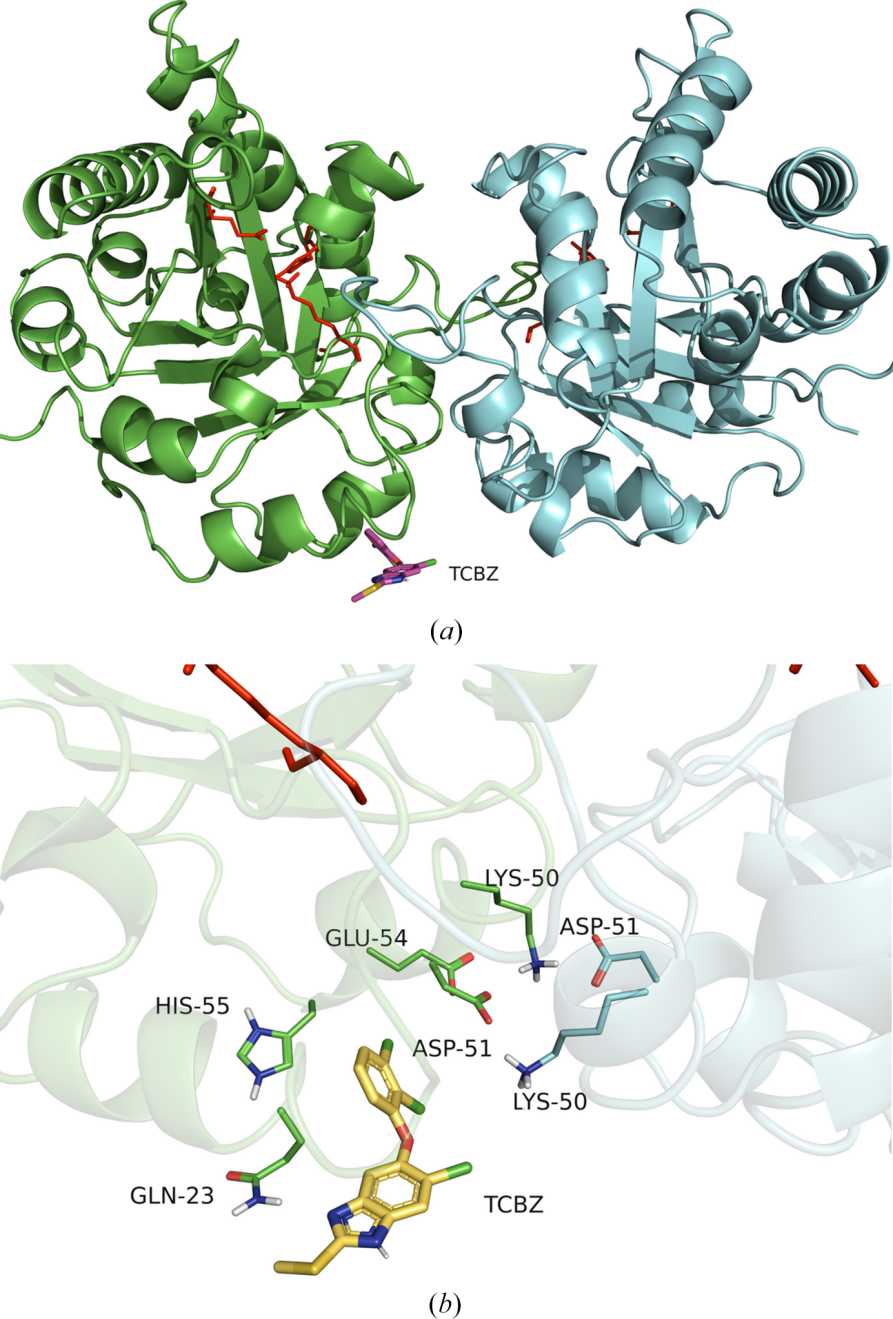
(*a*) Front view of the *Fh*TPI dimer with the best docking pose of TCBZ (magenta) displayed. The dimer interface was used as the docking area. Active-site residues for each monomer are shown as red sticks. (*b*) Close-up view of the best docking pose showing TCBZ (magenta) docked close to the *Fh*TPI dimer interface. Residues Lys50 and Asp51 (participating in the dimer interface) and residues within a radius of 4 Å from TCBZ are displayed. Active-site residues for each monomer are shown as red sticks. This image was produced with *PyMOL* (Schrödinger).

The first experiment encompassed searching the entire dimer surface and produced ten docking poses in total, ranked by free binding energy. None of the docking poses was in proximity to the active-site residues. The first docking pose was proximate to the first α-helix (H1) close to the N-terminus (Fig. 5 ▸*a*), with a binding energy of −7.34 kcal mol^−1^, an inhibition constant of 4.17 µ*M* and no predicted hydrogen bonds formed between the receptor and ligand. The second (Fig. 5 ▸*a*) and third (not shown) poses have a similar binding energy to the first (−7.01 and −6.98 kcal mol^−1^, respectively) and are both located in the same position with a slightly different degree of rotation of the TCBZ oxygen bond. The inhibition constants are 7.31 and 7.59 µ*M* for the second and third pose, respectively. In the third pose, the hydrogen (HE21) of Gln23 is predicted to form a hydrogen bond to the nitrogen (N1) of TCBZ (Fig. 5 ▸*b*). Notably, in the position of Gln23, according to the performed multiple sequence alignment (Fig. 4 ▸), a glycine is found in the mammalian hosts. This could potentially mean that the binding affinity of TCBZ to mammalian TPI is different compared with fluke TPI. In the second docking experiment, the search area was restricted to the dimer interface. The best pose (Fig. 6 ▸*a*), as well as the second, third and fourth best from the total of ten (ranked by binding energy), docked in the same area as the second and third docking poses of the previous experiment searching the whole dimer surface. The first, second and third poses have binding energies of −6.8, −6.7 and −6.7 kcal mol^−1^ and inhibition constants of 9.7, 12.4 and 12.4 µ*M*, respectively. Additionally, the third pose of TCBZ is specifically predicted to form the same hydrogen bond as the third pose of the abovementioned ‘blind’ docking experiment. Remarkably, the poses are located (with different conformations) close to Lys50 and Asp51 (Fig. 6 ▸*b*). These two residues form two hydrogen bonds and three salt bridges (out of a total of seven) that participate in dimer-interface formation (as predicted by *PDBePISA*). In the mammalian hosts of *F. hepatica* (*Bos taurus*, *Ovis aries* and *H. sapiens*) they are substituted by Asp50 and Phe51 (Fig. 4 ▸), which do not form these interactions. In the hosts Phe51 is not predicted to participate in dimer-interface interactions and thus they lack the interactions of Lys50 and Asp51. Although the predicted inhibition constant for TCBZ is probably not adequate to disrupt the dimer interface considering the size of the interface, it could affect the dimerization process. This leads us to think and hypothesize that in *F. hepatica* these two residues are important in dimer formation and that TCBZ could interrupt the dimer formation or potentially change the conformation of the dimer, rendering it less active or inactive. This could be an indication of the selectivity of TCBZ for the fluke TPI and its role as an allosteric effector of *Fh*TPI. These findings in our opinion warrant experimental confirmation with functional studies. Overall, this structure may lead to subsequent studies aiming to identify molecular drug targets on *Fh*TPI and consequently the design of improved fasciolocides.

## Supplementary Material

PDB reference: monoclinic triosephosphate isomerase from *Fasciola hepatica*, 7qon

## Figures and Tables

**Table 1:** Data collection, processing, structure solution and refinement of *Fh*TPI Values in parentheses are for the highest resolution shell.

Data collection	
Beamline	I02, Diamond Light Source
Wavelength (Å)	0.9795
Space group	*P*2_1_
*a*, *b*, *c* (Å)	42.4, 110.5, 118.1
α, β, γ (°)	90.0, 97.4, 90.0
Resolution range (Å)	51.76–1.51 (1.54–1.51)
Total reflections	537159 (27159)
Unique reflections	167030 (8244)
Completeness (%)	99.1 (99.7)
Multiplicity	3.2 (3.3)
*R*_meas_† (%)	7.1 (173.6)
〈*I*〉/〈σ(*I*)〉	10.9 (0.8)
CC_1/2_‡	0.999 (0.321)
Wilson *B* factor§ (Å^2^)	29.6
Model refinement	
*R*_work_	0.165
*R*_free_	0.193
Monomers in asymmetric unit	4
No. of atoms	
Protein	8463
Solvent	1164
No. of residues	1006
R.m.s.d., bond lengths (Å)	0.008
R.m.s.d., bond angles (°)	1.41
Ramachandran plot favoured¶ (%)	97.5
Ramachandran plot outliers¶ (%)	0.40
Clashscore¶	6.79
Average *B* factor, protein (Å^2^)	28.5
Average *B* factor, solvent (Å^2^)	40.8
PDB code	7qon

† *R*_meas_ = 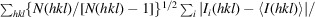

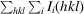
.

‡ CC_1/2_ is defined in Karplus & Diederichs (2012 ▸).

§ The Wilson *B* factor was estimated by *SFCHECK* (Vaguine *et al.*, 1999 ▸).

¶ The Ramachandran statistics and clashscore were calculated using *MolProbity* (Williams *et al.*, 2018 ▸).
